# Effect of development speed and quality of blastocyst on singleton birthweight in single frozen-thawed blastocyst transfer cycles

**DOI:** 10.3389/fendo.2023.1307205

**Published:** 2024-01-15

**Authors:** Xue Wang, YaLing Xiao, ZhengYi Sun, Tao Tao

**Affiliations:** Department of Gynecology Endocrine and Reproductive Center, State Key Laboratory of Complex Severe and Rare Disease, Peking Union Medical College Hospital, Chinese Academy of Medical Sciences and Peking Union Medical College, Beijing, China

**Keywords:** blastocyst quality, blastocyst development speed, FBT, singleton, birthweight

## Abstract

**Background:**

Assisted reproductive technology (ART) has revolutionized infertility treatment, leading to a surge in ART-conceived children. Despite its success, ART-born offspring face higher risks of preterm birth (PTB), low birth weight (LBW), and small for gestational age (SGA). The mechanisms behind these outcomes remain unclear, partly attributed to multiple embryo transfers. Recent advancements advocate single blastocyst transfers for improved outcomes. However, the influence of blastocyst quality and development speed on neonatal outcomes is underexplored.

**Objective:**

This study investigated whether blastocyst development speed and quality affect singleton birthweight when the blastocyst is selected for single frozen-thawed blastocyst transfer (FBT).

**Methods:**

Data from patients who performed an FBT cycle at our center from July 2011 to June 2021 were collected and analyzed. Based on the inclusion and exclusion criteria, 420 single FBT cycles were assessed. The women were divided into four groups, Group A (day 5, good-quality blastocysts), Group B (day 5, non-good-quality blastocysts), Group C (day 6, good-quality blastocysts), and Group D (day 6, non-good-quality blastocysts) according to the developmental speed and quality of the transferred blastocyst.

**Results:**

The birthweight was relatively the highest in Group A, which developed rapidly and transferred good quality blastocysts. However, no significant difference existed among the groups (*P*>0.05). The prevalence of premature birth (PTB), low birth weight (LBW), very low birth weight (VLBW), or high birth weight (HBW) was similar among the four groups (*P* > 0.05). No correlation existed between birth weight and blastocyst development speed or quality after adjusting for possible confounders (*P* > 0.05 respectively). However, the difference in the proportion of males born among the four groups was significant, especially in Group D, which was significantly lower than that in Group A (adjusted odds ratio = 0.461, 95% confidence interval: 0.230–0.921, *P* < 0.05).

**Conclusions:**

This retrospective cohort study suggests that the combined effect of blastocyst development speed and quality on neonatal birthweight is insignificant. The transfer of slow-growing, non-good-quality blastocysts increases the chance of a female baby being born.

## 1 Introduction

Assisted reproductive technology (ART) has become an effective means of treating infertility, with more children delivered through it (1, 2). Many studies have observed that children born through ART have an increased risk of PTB, LBW, and SGA compared to those born naturally (3, 4); therefore, the safety of ART-born children has also gained attention (2, 5). However, the related mechanisms causing the poor neonatal outcomes remain unclear. A crucial reason is the transfer of several embryos to obtain a good clinical pregnancy outcome, resulting in a higher multiple pregnancy rate and adverse birth outcomes (6). In recent years, with improvements in ART, the pregnancy rate has dramatically improved; thus, many centers have begun to reduce the number of embryos transferred, including single embryos (7). Studies have confirmed that the clinical outcomes after blastocyst transfer are higher than those after cleaved embryo transfer, and single-blastocyst transfer is recommended (6, 8). Therefore, birth outcomes of single blastocyst transfers have gained attention.

Selecting embryos for blastocyst transfer is based on blastocyst morphological evaluation because blastocyst quality plays a crucial role in determining pregnancy outcomes (9, 10). However, only a few studies have focused on the effect of blastocyst quality on neonatal birthweight, and their conclusions have been inconsistent (11–14). In addition, the blastocyst development speed is another criterion for embryo selection (15). The developmental rate of blastocysts of early human embryos varies from day 5 (D5) to day 7 (D7) after fertilization (16, 17). Most studies have reported that faster blastocyst development is positively related to higher developmental potential (16, 18). However, there are little data on the effect of blastocyst development speed on neonatal birth weight. Only two recent studies have observed no correlation between blastocyst development speed and neonatal outcomes, such as premature delivery and birthweight (19, 20). However, when deciding which blastocyst to transfer in clinical practice, the quality of blastocysts and their development speed should both be considered. Therefore, analyzing the simultaneous influence of blastocyst development speed and quality on the neonatal birth outcome is essential. The uterus is in a high-estrogen environment during fresh cycle transplantation, which may lead to abnormal changes in the endometrium, possibly affecting embryo implantation and placental development, inducing a series of challenges, such as low birth weight (21, 22). Therefore, the women who underwent a single FBT were included in the study.

The birth weight of newborns is an indicator of their health. Infants that are SGA or large for their gestational age (LGA) are more likely to have cardiometabolic abnormalities in childhood and adolescence, resulting in the risk of cardiovascular disease and obesity increasing in the future (23, 24). Therefore, in this study, we attempted to analyze the effects of blastocyst development speed and quality on singleton birthweight when selecting embryos for single FBT.

## 2 Materials and methods

### 2.1 Study design and patients

The data of women who underwent FBT cycles at the Gynaecological Endocrinology and Assisted Reproduction Centre of Peking Union Medical College Hospital from July 2011 to June 2021 were retrospectively analyzed. The data of patients who met the inclusion criteria were included in the analysis. The following were the inclusion criteria: (1) age < 40; (2) single FBT; (3) singleton delivery; (4) blastocyst stage of four. The exclusion criteria included the following: (1) congenital uterine malformation, including unicornuate uterus; (2) patients with conditions such as hypertension, diabetes, liver disease, and thyroid dysfunction; (3) patients with a history of tumors, such as endometrial cancer; (4) vanishing twin syndrome; (5) gestational diabetes, pregnancy-induced hypertension, and eclampsia; (6) preimplantation genetic testing cycles; (7) repeated cycles. The study was approved by the Ethics Committee of the Peking Union Medical College Hospital. Due to the study’s retrospective nature, the requirement for informed consent was waived. Based on the development speed and quality of the blastocysts transferred, the included women were divided into four groups, i.e., Group A (D5, good-quality blastocysts), Group B (D5, non-good-quality blastocysts), Group C (D6, good-quality blastocysts), and Group D (D6, non-good-quality blastocysts).

### 2.2 Treatment protocol and embryo evaluation

Gonadotropin-releasing hormone (GnRH) agonists or antagonists were used for pituitary suppression before ovarian stimulation at our center. The follicle-stimulating hormone was used for ovarian stimulation. When more than two dominant follicles reached 18 mm in diameter, 250 μg human chorionic gonadotrophin (HCG, Serono, Switzerland) was injected to stimulate the last maturation of the oocyte, and the oocytes were retrieved 38 h later. Then fertilization was performed according to the semen of her husband. On day 3 (D3), after fertilization, the embryos were scored and selected for transfer. With the patient’s informed consent, all remaining embryos were cultured until D5 or D6 after fertilization. Upon formation of blastocysts, they were accessed in accordance with the Gardner scoring system (25). In brief, blastocysts were scored based on three aspects, i.e., blastocyst cavity expansion (1–6), the quality of the inner cell mass (ICM) (A, B, C), and the quality of trophoblasts (TE) (A, B, C). Based on our experience, we found the recovery rates of early blastocysts at stage 3 or earlier were not ideal, so we selected blastocysts at stage 4 or above for cryopreservation. In addition, the embryos were observed twice daily, in the morning and afternoon; therefore, most cryopreserved blastocysts were at stage 4. The vitrification criteria used for blastocysts were that the ICM must be grade A or B and the TE must be grade A, B, or C. Blastocysts with ICM and TE of grade A or B, i.e., AA, AB, BA, and BB, were defined as good-quality and BC blastocysts were defined as non-good-quality blastocysts.

### 2.3 Cryopreservation of blastocysts and endometrial preparation

Prior to vitrification freezing, all the blastocysts underwent artificial shrinking using a laser. Then, the blastocysts were equilibrated in a pre-equilibration solution containing dimethyl sulfoxide (DMSO) and ethylene glycol for 2 min, transferred to a vitrification solution for 30 s, transferred to a cryotop (Kato, Japan), and immersed in liquid nitrogen for preservation directly. The frozen blastocysts were thawed on the day of the FBT. Moreover, all the thawed blastocysts underwent laser hatching, far from the ICM, and the zona pellucida was cut approximately by one-quarter. Then, the thawed blastocysts were transferred to the blastocyst culture medium (G2, Vitrolife, Sweden) prepared one day prior and cultured in an incubator at 37 °C, 6% CO_2,_ and 5% O_2_ for approximately 2 h.

Endometrial preparation and luteal support protocols for FBT cycles at our center have been described in a previous article (26). In summary, two approaches to endometrial preparation exist. The natural cycle method applies to patients with regular menstrual cycles and normal ovulation. The artificial cycle method is suitable for patients with irregular menstruation. When the thickness of the endometrium reached 8 mm, progesterone (Crinone; Merck Serono, Feltham, UK) was administered intramuscularly or vaginally, and the blastocyst was transferred on the sixth day. Luteal support was routinely provided by the intramuscular or vaginal administration of progesterone after transfer.

### 2.4 Outcome definitions

The main outcome measure was singleton birth weight, which was weighed by two experienced doctors and nurses immediately after birth. Usually, the newborns are measured by the baby scales that meet the national measurement standards: accurate to G, and the maximum weighing weight is generally not more than 15kg. When measuring, the child can be placed in the center of the weighing pan to read the weight of the child. Other neonatal outcomes included PTB, gestational age of 28 weeks and < 37 weeks; VPTB, gestational age of < 32 weeks; LBW, birthweight < 2500 g; VLBW, referred to birthweight < 1500 g; and macrosomia, birthweight ≥ 4000 g. SGA is also the 10th percentile birth weight lower than the average for the same gestational age; LGA was the 90th percentile birthweight above the mean weight for gestational age.

### 2.5 Statistical analysis

All data were analyzed using SPSS 22.0 software (Statistical Package for Social Sciences, IBM). The basic data and birth outcomes were compared among the groups. When the data are continuous variables, a normality test is performed. If the variables meet normality, they were analyzed using one-way analysis of variance (ANOVA); otherwise, non-parametric testing is required. While categorical data were analyzed using the chi-square or Fisher’s exact test. The effects of blastocyst quality and development speed on birth outcomes were assessed using a multivariate logistic model and expressed as crude odds ratios (ORs) and adjusted ORs with 95% confidence intervals (CI). The influence of blastocyst quality and development speed on birthweight was analyzed using multivariate linear regression. The adjusted potential confounders included infertility style, maternal BMI, maternal age, infertility duration, the reason for IVF, parity, endometrial preparation protocol, fertilization method, the thickness of endometrial, and ovulation promotion protocol. Group A was the control. *P* < 0.05 was considered statistically significant.

## 3 Results

A total of 420 patients were eligible for enrolment in this study. The baseline characteristics of the women in each group are presented in **Table 1**. Both the maternal age and BMI showed no significant difference among the groups. The duration, and types of infertility showed substantial differences among the four groups (*P* < 0.05). The incidence of male factors was higher in group D, resulting in a slightly higher proportion of intracytoplasmic sperm injection than in the other groups. However, no significant differences were found in the protocol for ovulation induction, fertilization of fresh cycles, parity, endometrial thickness, or endometrial preparation protocol during resuscitation and transplantation among the four groups.

**Table 1:** Comparison of general characteristics of four groups of patients with different blastocyst developmental speed and embryo quality.

Items	Group A (n = 243)	Group B (n = 51)	Group C (n = 76)	Group D (n = 50)	*P*-value
**Female age (year)**	33.2 ± 3.7	34.2 ± 3.7	33.3 ± 4.0	34.1 ± 3.8	0.199
**BMI (kg/m^2^)**	21.2 ± 2.0	21.4 ± 1.9	21.1 ± 2.1	21.0 ± 1.9	0.814
**Infertility duration (years)**	4.2 ± 2.8	5.3 ± 2.7	4.8 ± 3.1	5.1 ± 3.0	0.03
Infertility etiology					
Endometriosis	68 (28.0%)	14 (27.5%)	17 (22.4%)	13 (26.0%)	0.811
Abnormal ovulation	92 (37.9%)	20 (39.2%)	24 (31.6%)	7 (14%)	0.010
Tubal factors	67 (27.6%)	23 (45.1%)	22 (28.9%)	17 (34.0%)	0.092
Male factors	110 (45.3%)	24 (47.1%)	36 (47.4%)	28 (56.0%)	0.589
Others	9 (3.7%)	1 (1.7%)	1 (1.3%)	3 (6.0%)	0.482
**Type of infertility**					0.026
Primary	162 (66.7%)	24 (47.1%)	48 (63.2%)	26 (52.0%)	
Secondary	81 (33.3%)	27 (52.9%)	28 (36.8%)	24 (48.0%)	
Parity					
0	220 (90.5%)	47 (92.2%)	68 (89.5%)	43 (86.0%)	0.740
>1	23 (9.5%)	4 (9.8%)	8 (10.5%)	7 (14.0%)	
**Ovulation stimulation regimen**					0.104
GnRHa	148 (60.9%)	34 (66.7%)	58 (76.3%)	33 (66.0%)	
GnRHan	95 (39.1%)	17 (33.3%)	18 (23.7%)	17 (34.0%)	
**Fertilization method**					0.781
IVF	144 (59.3%)	34 (66.7%)	46 (60.5%)	29 (58.0%)	
ICSI	99 (40.7%)	17 (33.3%)	30 (39.5%)	21 (42.0%)	
**Endometrial preparation**					0.231
Natural cycle	72 (29.6%)	12 (23.5%)	15 (19.7%)	17 (34.0%)	
Artificial cycle	171 (70.4%)	39 (76.5%)	61 (80.3%)	33 (66.0%)	
**Endometrial thickness (mm)**	10.5 ± 1.2	10.5 ± 1.1	10.6 ± 1.1	10.6 ± 1.1	0.972

BMI, body mass index; IVF, in vitro fertilization; ICSI, intracytoplasmic sperm injection; GnRHa, gonadotropin-releasing hormone agonist; GnRHan, gonadotropin-releasing hormone antagonist. P < 0.05 was considered statistically significant. The percentage was shown within the brackets.

The comparison of the outcomes of infants among the groups is shown in **Table 2**. No significant difference existed in the birthweight of singletons in each group (3382.1 ± 489.2 g, 3274.0 ± 736.0 g, 3290.2 ± 483.9 g, 3258.8 ± 475.7 g for Groups A, B, C and D, respectively, *P* > 0.05). However, the birthweight was the highest in Group A (with rapid development and good-quality embryos transferred) and lowest in Group D (with slow development and non-good-quality embryos transferred). Additionally, no significant difference existed among the groups in neonates with LBW, VLBW, HDW, LGA, and SGA (*P* > 0.05). However, a significant difference existed in the sex ratio among the four groups (*P* < 0.05), with the highest proportion of males (58.8%) in Group A and the lowest in Group D (36.0%).

**Table 2:** Comparison of neonatal outcomes in the four groups of patients with different blastocyst development speed and quality.

Items	Group A (n = 243)	Group B(n = 51)	Group C(n = 76)	Group D(n = 50)		*P*-value
**Mode of delivery**						0.959
Vaginal delivery	91 (37.4%)	18 (35.3%)	26 (34.2%)	18 (36.0%)		
Cesarean section	152 (62.6%)	33 (64.7%)	50 (65.8%)	32 (64.0%)		
**Gestational age**	274.3 ± 11.2	269.1 ± 18.9	272.6 ± 13.5	273.2 ± 10.4		0.067
**PTB**	15 (6.2%)	7 (13.7%)	7 (9.2%)	2 (4.0%)		0.193
**VPTB**	2 (0.8%)	1 (2.0%)	1 (1.3%)	0 (0%)		0.758
**Length at birth (cm)**	50.1 ± 2.0	49.4 ± 2.9	49.8 ± 2.2	49.7 ± 1.4		0.082
**Birthweight (g)**	3382.1 ± 489.2	3274.0 ± 736.0	3290.2 ± 483.9	3258.8 ± 475.7		0.235
**HBW**	23 (9.5%)	7 (13.7%)	2 (2.6%)	3 (6.0%)		0.115
**LBW**	9 (3.7%)	5 (9.8%)	2 (2.6%)	3 (6.0%)		0.208
**VLBW**	0 (0%)	1 (2.0%)	1 (1.3%)	0 (0%)		0.179
**LGA(> 90th percentile)**	64 (26.3%)	12 (23.5%)	16 (21.1%)	10 (20.0%)		0.681
**SGA(< 10th percentile)**	10 (4.1%)	2 (3.9%)	1 (1.3%)	1 (2.0%)		0.625
**Sex**						0.025
Male	143 (58.8%)	25 (49.0%)	41 (53.9%)	18 (36.0%)		
Female	100 (41.2%)	26 (51.0%)	35 (46.1%)	32 (64.0%)		

LBW, low birthweight; VLBW, very low birth weight; HBW, high birthweight; PTB, preterm birth; VPTB, very preterm birth; LGA, large for gestational age; SGA, small for gestational age; P < 0.05 was considered statistically significant. The percentage was shown within the brackets.

Multivariate logistic regression analysis showed no significant differences existed in neonates with PTB, VPTB, LBW, VLBW, SGA, or LGA among the groups (*P* > 0.05). After adjusting for confounding factors, the probability of PTB in Group B was 3.680 times higher than in Group A (*P* < 0.05). However, no significant differences existed in the other comparisons (**Table 3**). VLBW and VPTB have also been analyzed; nonetheless, they had low incidence, and the data are too sparse to be meaningful. However, a significant difference existed in the proportion of males born, especially in Group D, which was significantly lower than in Group A (adjusted odds ratio = 0.461, 95% confidence interval: 0.230–0.921, *P* < 0.05). The multiple linear regression analysis of the development speed and quality of singleton birthweights is presented in **Table 4**, which shows no correlation existed between birthweight and blastocyst development speed and quality (Group B compared with Group A, β:-3.019, *P* = 0.934; Group C, β:-21.578, *P* = 0.340; Group D, β:-22.322, *P* = 0.209).

**Table 3:** Comparison of the relationship between blastocyst development speed and quality and neonatal outcomes.

Items	Group A	Group B	Group C	Group D
PTB				
Crude OR (95% CI)	Reference	2.418 (0.932–6.274)	1.542 (0.604–3.935)	0.633 (0.140–2.861)
Adjust OR (95% CI)	Reference	**3.680 (1.079**–**12.550)**	1.288 (0.335–4.951)	0.660 (0.108–4.017)
VPTB				
Crude OR (95% CI)	Reference	2.410 (0.214–27.094)	1.607 (0.144–17.968)	NA
Adjust OR (95% CI)	Reference	NA	NA	NA
LBW				
Crude OR (95% CI)	Reference	2.826 (0.906–8.819)	0.703 (0.149–3.325)	1.660 (0.433–6.361)
Adjust OR (95% CI)	Reference	3.431 (0.730–16.129)	0.247 (0.017–3.671)	0.964 (0.119–7.798)
HBW				
Crude OR (95% CI)	Reference	1.522 (0.615–3.765)	0.259 (0.060–1.123)	0.611 (0.176–2.118)
Adjust OR (95% CI)	Reference	1.356 (0.445–4.129)	0.392 (0.085–1.810)	0.457 (0.097– 2.161)
LGA				
Crude OR (95% CI)	Reference	0.861 (0.424–1.745)	0.746 (0.401–1.388)	0.699 (0.330–1.479)
Adjust OR (95% CI)	Reference	0.713 (0.316–1.610)	0.628 (0.280–1.412)	0.559 (0.238–1.310)
SGA				
Crude OR (95% CI)	Reference	0.951 (0.202–4.477)	0.311 (0.039–2.467)	0.476 (0.059–3.801)
Adjust OR (95% CI)	Reference	1.523 (0.200–11.572)	NA	NA
Male baby				
Crude OR (95% CI)	Reference	0.672 (0.367–1.232)	0.819 (0.488–1.376)	**0.393 (0.209**–**0.740)**
Adjust OR (95% CI)	Reference	0.604 (0.302–1.208)	0.994 (0.516–1.915)	**0.461 (0.230**–**0.921)**

LBW, low birthweight; HBW, high birthweight; PTB, preterm birth; VPTB, very preterm birth; LGA, large for gestational age; SGA, small for gestational age; OR, odds ratio; CI, confidence interval. NA, not available. Boldened values are significant.

**Table 4:** Multiple regression analysis of singleton birth weight.

Items	β	95.0% CI		P-value
		Lower	Upper	
Development speed and quality of blastocyst				
D5 high-quality blastocyst (reference)				
D5 poor-quality blastocyst	-3.019	-74.735	68.698	0.934
D6 high-quality blastocyst	-21.578	-66.036	22.881	0.340
D6 poor-quality blastocyst	-22.322	-57.229	12.585	0.209
Maternal age	9.304	-3.942	22.549	0.168
Endometrial thickness (mm)	16.717	-22.518	55.953	0.403
BMI (kg/m^2^)	1.779	-20.015	23.573	0.873
Endometrial preparation (compared with the natural cycle)	8.791	-97.621	115.204	0.871
Fertilization method (compared with IVF)	17.541	-105.774	140.856	0.780
Ovulation stimulation regimen (compared with GnRHa)	-83.654	-177.235	9.928	0.080
Type of infertility (compared with primary)	95.690	-8.433	199.813	0.072
Parity (compared with 0)	-27.612	-125.786	70.562	0.580
Infertility duration (years)	6.940	-9.427	23.308	0.405
Tubal factors	19.303	-90.647	129.254	0.730
Endometriosis	-33.563	-128.737	61.611	0.488
Male factors	-43.018	-167.726	81.689	0.498
Abnormal ovulation	-100.890	-186.959	-14.821	0.022
Mode of delivery(compared with CS)	-112.591	-206.017	-19.166	0.018
Gestational age	23.452	19.822	27.082	0.000
Sex of baby (compared with male)	138.606	48.790	228.422	0.003

BMI, body mass index; CS, Cesarean section; IVF, in vitro fertilization; GnRHa, gonadotropin-releasing hormone agonists. P < 0.05 was considered statistically significant.

## 4 Discussion

Many studies have focused on the influence of embryo quality or development speed on clinical pregnancy outcomes; however, fewer studies have been performed on the impact of birth weight. Herein, we investigated whether blastocyst development speed and blastocyst quality affected singleton birthweight in single FBT cycles.

The results are similar to those of several recent studies (19, 27, 28). He et al. (28) divided their study patients into two groups based on age (< 35 years and ≥ 35 years). They observed that the neonatal outcomes of the group with transferred D5 non-excellent embryos were similar to those of the group with transferred D6 excellent embryos in both age groups. Recently, a large retrospective study analyzed the effect of blastocyst development rate on neonatal weight (20). They revealed that the birthweight of the D5 group was higher than that of the D6 group. However, after adjusting for possible confounders, the regression analysis revealed no significant difference in birth weight between both groups. Thus, delayed blastocyst formation does not affect neonatal weight in the FET cycle. This could have been because the developmental potential of the blastocysts was similar once they were implanted despite their different development speeds (20). This is inconsistent with some conclusions of previous studies that slow blastocyst development affects neonatal outcomes (29, 30). In addition to lower pregnancy and live birth rates, the transfer of slow-growing D6 blastocysts results in a higher birthweight than D5 blastocysts, and neonates are more prone to LGA (30). However, the mechanism by which blastocyst development rate affects birthweight remains unclear. Animal studies have revealed that changes in the distribution of the TE and ICM during blastocyst formation can affect the fetal size. Moreover, when there are more TE cells, the placenta’s size increases. Contrarily, the fetus grows bigger when ICM cells increase (31, 32). Other studies have revealed that the composition of the culture medium may be responsible for differences in neonatal weight (33–35). Sander et al. (34) observed that different culture media result in significant differences in the birthweight of newborns and considered that embryos were very sensitive to the culture environment during *in vitro* culture. Caitlin et al. (35) observed that LGA was more likely to occur in a one-step culture than in a sequential culture, suggesting that culture media could affect neonatal outcomes. However, randomized studies on human embryos have revealed that the birthweight of newborns is unaffected by the culture medium’s composition (36). Whether the speed of blastocyst development affects birthweight requires further investigation.

A correlation exists between blastocyst quality and development speed. Generally, compared with low-quality embryos, the better-quality embryo was associated with a shorter time required to form blastocysts. Therefore, theoretically, transferring blastocysts with fast development and good quality will lead to better birth outcomes; however, this study did not find differences among the four groups. This is similar to the conclusions of some studies. For example, a study revealed that the gestational age and birthweight of low-quality blastocysts transferred on D5 did not significantly differ from those of high-quality blastocysts transferred on D6 (28). A large retrospective study, including data on 7,469 females with singleton births, revealed that both groups had similar outcomes after transferring a high- or non-high-quality blastocyst (14). Similarly, a recent large multicenter European study revealed no correlation between blastocyst quality and neonatal birthweight (19). However, this is contrary to the conclusion of Zhang et al. (37), who found that, compared with the high-quality blastocysts transferred cycles, the transfer of low-quality blastocysts resulted in a lower birthweight, and the embryo was more prone to SGA. Furthermore, they believed that this may be related to uterine factors. Biological signals in the endometrium that can identify embryo quality exist, affecting the secretion of specific growth factors and subsequently affecting placentation and fetal development (38). In addition, Xie et al. (39) observed that the birth weight of the fetus was positively related to the quality of trophoblasts; better blastocyst quality correlated with a higher singleton birth weight. Therefore, transferred blastocysts with a good-quality trophoblastic layer are more likely to have LGA, which better verifies the above hypothesis that the quality of TE cells is crucial to the quality of the placenta. The placenta is closely associated with neonatal health and birthweight (40). Similarly, when analyzing the relationship between trophoblasts of blastocysts and birthweight, the authors observed that grade C blastocysts were more likely to develop SGA than blastocysts with grade A trophoblasts (14). Thus, whether blastocyst quality affects singleton birthweight remains controversial. The differences between these studies may be related to the selection of the population and the definition of blastocyst quality. For example, blastocysts were scored more strictly at our center in this study. In 2005, we cultured blastocysts and observed that the pregnancy rate was very low when the ICM was of grade C; therefore, grade C blastocysts were discarded. In addition, to avoid the blastocyst stage’s influence on this study’s results, we included only stage 4 blastocysts. Whether blastocyst quality affects singleton birthweight requires verification in larger population studies.

In this study, we found that the transfer of slow-growing, non-good-quality blastocysts increased the chance that the baby born would be female. Multivariate regression analysis revealed that compared with Group A, the probability of male birth was significantly lower in Group D. This is inconsistent with the conclusions of some studies on the effect of blastocyst development speed on sex (19). Borgstrøm et al. (20) observed that the blastocyst development speed did not correlate with the sex ratio of the children. Similarly, Lou et al. (41) did not reveal that blastocyst development speed affected the sex of the newborn. In a recent meta-analysis, Zeng et al. (42) observed that the proportion of male newborns after frozen-thawed blastocyst transfer of D6 and D5 was similar. This may be because our comparison took into consideration the development speed and quality of the blastocysts. Based on our data, the proportion of males born from D6 suitable blastocysts was slightly higher than that from D5 non-good blastocysts. However, at the same development stage, the male birth rate (58.8% and 53.9%) of the group with good-quality embryos of D5 or D6 was higher than that of the group with non-good-quality embryos of D5 or D6 (49.0% and 36.0%, respectively), indicating that the delayed formation of blastocysts had a little effect on the sex. However, the quality of blastocysts was crucial to the sex ratio of the newborns. This finding is consistent with the conclusions of some studies. In a retrospective study of 7,469 females, the proportion of females born was significantly higher in those with non-good blastocyst transfer than that in those with good-quality blastocyst transfer. Moreover, the trophoblast quality of blastocysts transferred from women who had females born was poorer (*P* < 0.05) (14). In a recent multicenter European study of 4,842 live singleton births, regression analysis of birth sex revealed a 31% decrease in the likelihood of a male born from women with a grade B trophoblast blastocyst transfer compared to a grade A trophoblast blastocyst transfer (19). Similarly, Lou et al. (41) revealed that the probability of having a male baby after a high-quality single blastocyst transfer was significantly higher than that after a low-quality single blastocyst transfer (60% vs. 49.7%, *P* < 0.001). The reason might be that male embryos develop faster and have better morphological scores than female embryos at the same development stage (43). In clinical practice, selecting good-quality blastocysts for cryopreservation will be prioritized. We will also prioritize the selection of high-quality blastocysts frozen on D5 for FET to ensure that patients become pregnant promptly. This may have also led to a larger proportion of male embryos in the D5 good-quality blastocyst group. The proportion of male embryos in the non-good-quality D6 blastocysts was the lowest, consistent with a previous explanation; however, the specific mechanism requires further study.

This study aimed to analyze whether blastocyst selection that combined blastocyst development speed and quality in FET cycles affected singleton birth weight. However, this study had some limitations. First, due to the nature of this retrospective study, some confounding factors which could affect the birth weight of newborns, such as maternal nutritional status, regional distribution, education level, and economic conditions could not be obtained. Second, blastocyst evaluation has a certain degree of subjectivity. Blastocyst was evaluated by embryologists with substantial experience; nonetheless, some bias was inevitable. Moreover, the number of patients included was small, especially the number of non-good-quality blastocyst transfers. Lastly, to ensure the clinical pregnancy rate, a good-quality blastocyst was prioritized for transfer to patients, and the blastocyst was not randomly selected for transfer. In the future, multicenter cooperation should be considered for large-scale relevant data analyses. Finally, in this study, only patients < 40 years were included; therefore, the conclusions may not apply to older women.

## 5 Conclusions

This study suggests that selecting a single blastocyst for transfer, combined with blastocyst development speed and quality, has no significant effect on singleton birth weight. The transfer of slow-growing, non-good-quality blastocysts increases the chance of a female baby being born. However, the study results must be verified in a large-scale, randomized, controlled, multicenter study.

## Data availability statement

The original contributions presented in the study are included in the article/supplementary material. Further inquiries can be directed to the corresponding author.

## Ethics statement

The studies involving humans were approved by the Ethics Committee of the Peking Union Medical College Hospital. The studies were conducted in accordance with the local legislation and institutional requirements. Written informed consent for participation was not required from the participants or the participants’ legal guardians/next of kin in accordance with the national legislation and institutional requirements.

## Author contributions

XW: Data curation, Methodology, Writing – original draft, Writing – review & editing. YX: Data curation, Resources, Writing – review & editing. ZS: Software, Supervision, Writing – original draft. TT: Software, Writing – review & editing.
